# Genomic prediction using DArT-Seq technology for yellowtail kingfish *Seriola lalandi*

**DOI:** 10.1186/s12864-018-4493-4

**Published:** 2018-01-30

**Authors:** Nguyen H. Nguyen, H. K. A. Premachandra, Andrzej Kilian, Wayne Knibb

**Affiliations:** 1 The University of the Sunshine Coast, Maroochydore DC, QLD 4558 Australia; 2 Diversity Arrays Technology Pty Ltd, Kirinari St, Bruce, ACT 2617 Australia

**Keywords:** Kingfish, Genetic improvement, Genomic prediction, Genomic selection and genotype by sequencing

## Abstract

**Background:**

Genomic prediction using Diversity Arrays Technology (DArT) genotype by sequencing platform has not been reported in yellowtail kingfish (*Seriola lalandi*). The principal aim of this study was to address this knowledge gap and to assess predictive ability of genomic Best Linear Unbiased Prediction (gBLUP) for traits of commercial importance in a yellowtail kingfish population comprising 752 individuals that had DNA sequence and phenotypic records for growth traits (body weight, fork length and condition index). The gBLUP method was used due to its computational efficiency and it showed similar predictive performance to other approaches, especially for traits whose variation is of polygenic nature, such as body traits analysed in this study. The accuracy or predictive ability of the gBLUP model was estimated for three growth traits: body weight, folk length and condition index.

**Results:**

The prediction accuracy was moderate to high (0.44 to 0.69) for growth-related traits. The predictive ability for body weight increased by 17.0% (from 0.69 to 0.83) when missing genotype was imputed. Within population prediction using five-fold across validation approach showed that the gBLUP model performed well for growth traits (weight, length and condition factor), with the coefficient of determination (R^2^) from linear regression analysis ranging from 0.49 to 0.71.

**Conclusions:**

Collectively our results demonstrated, for the first time in yellowtail kingfish, the potential application of genomic selection for growth-related traits in the future breeding program for this species, *S. lalandi*.

**Electronic supplementary material:**

The online version of this article (10.1186/s12864-018-4493-4) contains supplementary material, which is available to authorized users.

## Background

Yellowtail kingfish (YTK) *Seriola lalandi* has emerged as an important marine finfish for aquaculture, not only in Australia but also in many other parts of the world including Japan, Latin America (Chile, Mexico), the United States and New Zealand [1]. In Australia, the hatchery and production technologies have been well established for this species, and a genetic improvement program has started since 2010, using the founder stocks collected from the wild in South Australia [2]. To date, the selection program for YTK has been practised solely for growth rate. Body condition of the fish was also assessed when selection decision was made because there is a demand for well conformed fish from overseas markets [3]. Genetic evaluation of the first two generations of family selection showed that there was a significant improvement in fry performance by approximately 8% per generation [2]. Performance testing of growers produced in the second generation (G2) in sea cages however showed lower magnitude of selection response when the selected fish were compared with wild stocks or with offspring of the G1 parents (Premachandra et al., submitted). The positive response to selection achieved in the present YTK population was consistent with those reported for other aquaculture species, with an average genetic gain ranging from 5 to 15% per generation [4–9]. In the YTK population of this study, moderate heritabilities (h^2^ = 0.15 to 0.41) were estimated for body and carcass traits [2, 3].

Along with the conventional selective breeding approach based on phenotype and pedigree, we are interested in exploring potential use of molecular and genomic information to accelerate genetic gain in this line of YTK, especially improving traits that are difficult or expensive to measure or lowly heritable. To date, high density SNP array-based chips are not commercially available for yellowtail kingfish *S. lalandi*. Further, the cost of whole genome sequencing is still high, especially for non-model species. Genotyping by sequencing (GBS) has been used as an alternative to whole genome sequencing approach due to its low cost of genotyping per animal, large amount of high quality genetic markers and the suitability of the markers for genomic prediction/selection. Among the Restricted-site Associated DNA sequencing (RAD-seq) methods, DArTseq™ sequencing technology represents a combination of a DArT complexity reduction methods and next generation sequencing platforms [10–13]. Similar to DArT methods based on array hybridisations, the technology is optimized for each organism and application by selecting the most appropriate complexity reduction method (both the size of the representation and the fraction of a genome selected for assays) and it can generate a large number of markers at a reasonable price per sample (approx. 35–50 AU$/sample by the time of this report). To the best of our knowledge, the DArTseq™ sequencing technology has not been reported for any aquatic species. However, many studies have applied variations of DArT genotyping by sequencing methods in aquaculture, including those used for genomic prediction. A synthesised result from the literature shows that there is potential for the application of genomic selection in salmonids [14, 15] or gilthead seabream [16]. To date, there is no published information whether DNA markers obtained from next generation sequencing platforms can be used to predict breeding values for complex traits in yellowtail kingfish (*S. lalandi*).

Therefore, the principal aim of this study was to investigate the predictive ability of genomic Best Linear Unbiased Prediction (gBLUP) method for commercial traits of economic importance in yellowtail kingfish (*Seriola lalandi*), using the DArT sequencing technology. It is the most common method used to perform genomic prediction for complex traits whose variation is of polygenic nature. Specifically, we report the gBLUP prediction for growth traits (body weight, fork length and condition index), using five-fold across validation conducted in the same population. The effects of the number of markers on the predictive ability of gBLUP model were also estimated.

## Methods

### Fish samples and phenotypic data

In this study, a total of 752 DNA samples of yellowtail kingfish (YTK) were sequenced using DArT-seq technology (see section “Library preparation and DArT sequencing”). The animals originated from a selective breeding program for YTK at Clean Sea Tuna Ltd. in South Australia. The fish samples analysed in this study were offspring of 35 families produced from the first generation (G1) and wild brood stock parents. The average number of offspring per family was 17. Breeding was conducted in tank, comprising three males and three females. In total, 16 sires and 31 dams successfully produced offspring in this study. After a nursing/rearing period of about 120 days in tanks, fingerlings were transferred to culture in sea cages. When the fish reached an average body weight of 3 kg, they were harvested and then anesthetised using clove oil (40 mg/L) and cold water before morphometric measurements and fin tissue collection. The morphometric and phenotypic measurements included body weight (W), fork length (L, measured from the tip of the snout to the end of the caudal fin rays). The condition index (factor) was calculated as k = W/L^3^. Deformities [17] included a range of measures, namely deformed snout, water belly – a condition where the belly is distended, deformed tail, deformed operculum and lower jaw). Skin fluke is due to the monogenean fluke parasite *Benedenia seriolae*; this fluke inhabits the skin and fins of *Seriola spp*. and feeds on mucus and epithelia cells. Both deformity and fluke were recorded as binary traits depending on their presence or absence on the body of the fish at harvest (~ 3 kg) and coded as 1 and zero, respectively. The incidence of skin fluke and deformity recorded under field condition in this population was low (4.3 and 17.6%, respectively) and hence, results from genomic prediction for these traits were not tabulated in this paper.

The fin caudal tissue sample was collected from caudal fin of each fish and stored in 80% ethanol for DNA extraction at a laboratory of the University of the Sunshine Coast (USC). Fish biometrics, fluke abundancy and deformities were recorded before they were released back to the cage.

A detailed description of the population is given in our earlier studies [2, 3, 17].

### DNA extraction, genotyping and parentage analysis

Fin tissue samples of a total of 1568 fish were used to extract total genomic DNA (gDNA) using DNeasy Blood and Tissue kits (Qiagen, Germany) and NucleoMag® 96 Tissue kit (MACHEREY-NAGEL GmbH & Co. KG, Germany). A panel of eight microsatellite markers that consisted of five newly developed candidates from *S. lalandi* transcriptome sequences (YTK002, YTK008, YTK011, YTK017 and YTK019) [3] and three loci (Sdu21, Sdu32 and Sdu46) validated from the literature [18, 19] were used to genotype the experiment fish for parentage analysis in this study. Genotyping the broodstock fish was completed in our previous study [2]. All of these markers were proved and validated to use with yellowtail kingfish previously [2, 3].

DNA amplification was achieved using Qiagen Multiplex PCR PLUS Kits (Qiagen, Germany) in 13.5 μL reactions, each containing 1.25 μL of 10× primer mix, 6.25 μL of Multiplex PCR Master Mix, 2.75 μL of RNase free water, 1.25 μL of Q-Solution and 2.0 μL of approximately 20 ng template gDNA. Amplification was performed using an Eppendorf Mastercycler nexus (Hamburg, Germany) with cycling conditions as follows: initial denaturation at 95 °C for 15 min, followed by 35 cycles of 95 °C for 30 s, 57 °C for 90 s, and 72 °C for 30 s; with a final extension at 68 °C for 30 min.

PCR products were separated by capillary electrophoresis on an AB 3500 Genetic Analyser (Applied Biosystems) at the University of the Sunshine Coast. Fragment sizes were determined relative to an internal lane standard (GS-600 LIZ; Applied Biosystems) using GENEMARKER v1.95 software (SoftGenetics; State College, USA) and double-checked manually. Individuals with low or missing peaks were amplified/genotyped a second time and checked for evidence of large allele dropout, extreme stuttering and null alleles, based on 1000 bootstraps and a 95% confidence interval. Tests for HWE at each locus and genotypic linkage equilibrium among pairs of loci, numbers of alleles and the observed and expected heterozygosities of each locus were determined using GENALEX v6.5, while polymorphic information content (*PIC*) was computed in CERVUS v3.0. Parentage assignment was completed using COLONY software [20] with confidence scores of above 95%. The pedigree included 65 full-sib groups (16 dams and 31 sires), with the family size of 3 to 108 offspring. A total of 1568 offsprings out of 1998 were assigned to full sib families and the family size. Based on this pedigree, large size families (35 full-sib families and averaging 17 fish per family) were chosen to send to DArT Ptd Ltd. in Canberra, Australia for sequencing.

### Library preparation and DArT sequencing

Four methods of DArTseq™ complexity reduction were tested in *Seriola lalandi* (data not presented) and the PstI-SphI method was selected. DNA samples were processed in digestion/ligation reactions principally as per Kilian et al. [10] but replacing a single PstI-compatible adaptor with two different adaptors corresponding to two different Restriction Enzyme (RE) overhangs. The PstI-compatible adapter was designed to include Illumina flowcell attachment sequence, sequencing primer sequence and “staggered”, varying length barcode region, similar to the sequence reported by Elshire et al. [21]. Reverse adapter contained flowcell attachment region and SphI-compatible overhang sequence.

Only “mixed fragments” (PstI-SphI) were effectively amplified in 30 rounds of PCR using the following reaction conditions: 94 °C for 1 min, then 30 cycles of 94 °C for 20 s, 58 °C for 30 s, 72 °C for 45 s and 72 °C for 7 min.

After PCR equimolar amounts of amplification products from each sample of the 96-well microtiter plate were bulked and applied to c-Bot (Illumina) bridge PCR followed by sequencing on Illumina Hiseq2500. The sequencing (single read) was run for 77 cycles.

Sequences generated from each lane were processed using proprietary DArT analytical pipelines. In the primary pipeline the fastq files were first processed to filter away poor quality sequences, applying more stringent selection criteria to the barcode region compared to the rest of the sequence (minimum Phred pass score = 30 and minimum pass percentage = 75). In that way the assignments of the sequences to specific samples carried in the “barcode split” step were very reliable. Approximately 2,500,000 sequences per barcode/sample were identified and used in marker calling. Finally, identical sequences were collapsed into “fastqcoll files”. The fastqcoll files were “groomed” using DArT PL’s proprietary algorithm which corrects low quality base from singleton tag into a correct base using collapsed tags with multiple members as a template. The “groomed” fastqcoll files were used in the secondary pipeline for DArT PL’s proprietary SNP and SilicoDArT (presence/absence of restriction fragments in representation) calling algorithms (DArTsoft14).

For SNP calling all tags from all libraries included in theDArTsoft14 analysis are clustered using DArT PL’s C++ algorithm at the threshold distance of 3, followed by parsing of the clusters into separate SNP loci using a range of technical parameters, especially the balance of read counts for the allelic pairs. Additional selection criteria were added to the algorithm based on analysis of approximately 1000 controlled cross populations. Testing for Mendelian distribution of alleles in these populations facilitated selection of technical parameters discriminating well true allelic variants from paralogous sequences. In addition, multiple samples were processed from DNA to allelic calls as technical replicates and scoring consistency was used as the main selection criteria for high quality/low error rate markers. Calling quality was assured by high average read depth per locus (Average across all markers was over 30 reads/locus). Regarding whole read quality, the minimum Phred pass score was set at 10 and the minimum pass percentage was 50. The average SNP calling rate was 92% and the fish sample calling rate was 95%. The raw sequence data (accession number SRP130211) is available at https://www.ncbi.nlm.nih.gov/sra/SRP130211 (Release date: 2019-02-28) Release date: 2019-02-28.

### GBS data analysis

Sample and markers statistics is given in Additional file 1: Tables S1 and S2, respectively. In total, there were 14,448 SNP markers. The PIC value for the SNPs was 0.16 under additive genetic model, whereas it was substantially higher, 0.46 under codominant model. The average proportion of missing genotype (SNPs) was only 14.8%. The frequency of minor allele was 0.29. The missing genotype was also imputed using theDArTsoft14 analysis pipeline.

### Pedigree based analysis of heritability

Restricted maximum likelihood (REML) method applied to a linear (animal) mixed model was used to estimate heritability for traits studied. The model included the fixed effect of stock origin (wild and F1 fish) and the additive genetic random effect of individual fish in the pedigree. Our preliminary analysis using logarithmic likelihood ratio test (LRT) indicated that the common full-sib effect was not significant (*P* > 0.05). This has been a result of early communal rearing of all families soon after birth.

Heritability (h^2^) for the traits studied was estimated as h^2^ = σ^2^_A_/σ^2^_P_ where σ^2^_A_ is the additive genetic variance, σ^2^_P_ the phenotypic variance (σ^2^_P_ = σ^2^_A_ + σ^2^_E_) and σ^2^_E_ is the residual variance. Pedigree based analysis of heritability were carried out using the ASREML software package [22].

### Genomic prediction

Genomic best linear unbiased prediction (gBLUP) method was used to assess the predictive ability of the model for traits studied, using SVS Suite (Golden Helix, 2016). This method (gBLUP) generally shows similar predictive capacity to other non-linear approaches, especially for growth traits as used in this study. Theoretical statistics of the gBLUP method was discussed in earlier reports [23]. In brief, the statistical model and assumptions regarding SNP distribution and its variance are given below.

The (gBLUP) method uses genomic relationship matrix (*G*) derived from DNA marker information to calculate genomic breeding values for each individual in the pedigree Clark et al., [24]. The model is written in a matrix notation as follows:

$$ y=m+ Xb+ Za+e $$

where ***y*** is the vector of phenotypic observations (body weight, length and condition index), ***m*** = overall mean, ***X*** is the incidence matrix containing fixed effects (i.e. stock origin in this study) in ***b***. The matrix ***Z*** relates records to genomic values, ***a*** is the SNP effect and ***e*** is the residual error. In this model, gBLUP assumes equal variance *σ*^2^_z_ for all loci/SNPs:

$$ Var(g)={ZZ}^{\prime }{\sigma^2}_z= G\phi \kern0.5em {\sigma^2}_z=G\kern0.5em {\sigma^2}_g $$

where *σ*^2^_g_ is the genetic variance and *ϕ* is the normalisation factor.

The pseudo heritability was calculated from the gBLUP model as ${h}_p^2={\widehat{\sigma}}_g^2$ /(${\widehat{\sigma}}_g^2$+${\widehat{\sigma}}_e^2$) where ${\widehat{\sigma}}_g^2$ is the genomic variance and ${\widehat{\sigma}}_e^2$ is the component of error variance.

The accuracy or predictive ability of the gBLUP model was defined as the correlations between the predicted breeding values and actual phenotypes (${r}_{y,\widehat{y}}$), divided by square root of the trait heritability.

### Within population prediction

We conducted within population prediction using five-fold cross validation approach to assess how well our model can predict the phenotype. With this approach, the training data consisting of 752 samples with both phenotype and genotype were randomly partitioned into five equal sub-samples (paternal half-sibs were present in all sub-sets), and in each round (e.g. k-1), one sub-set was selected as a test (validation) and the model fitted with four folds to predict the validation set.

The gBLUP five-fold cross validation model used was the same as described above. The allele substitution effects (ASE) and fixed effect coefficients obtained from iterations and k-folds that gave the largest R^2^-value were used to predict phenotypes with the following model:

$$ \widehat{y}=\mathbf{X}\widehat{\beta }+\mathbf{M}\widehat{\alpha } $$

where $\widehat{y}$ are the predicted phenotypes, ***X*** is the fixed effects matrix, $\widehat{\beta}$ are the fixed effect coefficients, ***M*** is the genotype matrix, and $\widehat{\alpha}$ are the ASE values.

The five-fold cross validation analyses for all the traits were conducted in SVS Suite (Golden Helix, 2016). We compared the actual and predicted phenotypes by association test and linear regression analysis. The coefficient of determination (R^2^) from the regression analysis (e.g. VanRaden et al. [25]) was also used to evaluate the predictive ability of the gBLUP model.

## Results

### Animals, phenotype and heritability

A total of 752 fish sampled from a population of 1568 individuals having phenotype data was sequenced using DArT marker technology. The number of observations, mean and standard deviation) for three traits studied are given in Table 1. Restricted maximum likelihood (REML) analysis applied to a single trait mixed model that included the fixed effect of stock origin and the additive genetic effect of individual animal as a random factor showed that the growth-related traits (i.e. body weight, fork length and condition index) were moderately heritable (h^2^ = 0.11 to 0.42).

**Table 1:** Phenotypic information of 752 fish sequenced and the heritability estimated from a pedigreed population (h^2^_a_ ± standard errors) and pseudo genomic heritability (h^2^_p_) for traits estimated from the markers, using gBLUP kinship matrix

Traits	Unit	N	Mean	SD	h^2^_a_ ± s.e.	h^2^_p_ ± s.e.
Weight	Kg	752	3.0	0.35	0.42 ± 0.10	0.47 ± 0.180
Length	Cm	752	58.2	2.10	0.42 ± 0.10	0.43 ± 0.230
Condition index	Unit	752	15.0	1.02	0.11 ± 0.05	0.21 ± 0.223

Pedigree based analysis of heritability was conducted on genotyped animals

*SD* Standard deviation

The pseudo-heritability (${h}_p^2$) of traits estimated from the marker data using gBLUP genomic relationship matrix is consistent with those estimated from the pedigree (Table 1). Growth related traits (weight, length and condition factor) had moderate pseudo-heritability (${h}_p^2$ = 0.21 to 0.47). Magnitudes of the pseudo-heritability estimates obtained from genomic data (SNPs) are generally similar among statistical methods used.

### Predictive ability of gBLUP model

The accuracy or predictive ability of genomic prediction for growth related traits (i.e. body weight, fork length and condition index) was moderate to high (0.44 to 0.69) (Table 2).

**Table 2:** Accuracy or predictive ability of gBLUP model for growth related traits

Traits		gBLUP
Weight		0.69
Length		0.67
Condition index		0.44

*gBLUP* Genomic best linear unbiased prediction

**Fig. 1:**
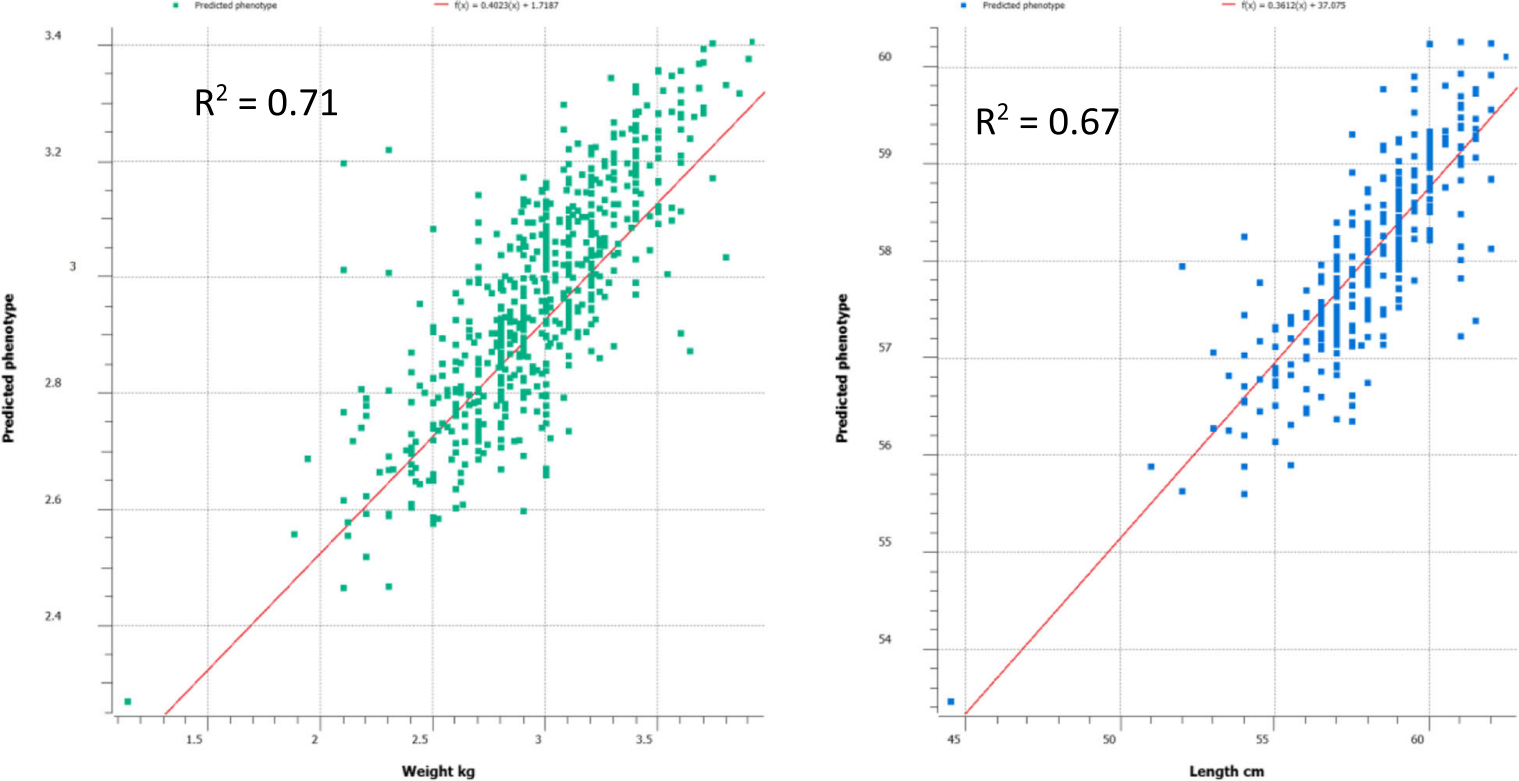
Five-fold across validation using gBLUP method for body weight and length

In addition, within population prediction using five-fold cross validation approach showed that the correlation between the predicted and actual phenotypes for all traits especially for weight and length were very high, ${r}_{y,\widehat{y}}$ = 0.76–0.89. The coefficient of determination (R^2^) from the linear regression analysis of the actual on predicted phenotypes ranged from 0.49 to 0.71 across the growth traits. The 5-fold cross validations using gBLUP for body weight and length are given in Fig. 1.

### Predictive ability using imputation data

**Fig. 2:**
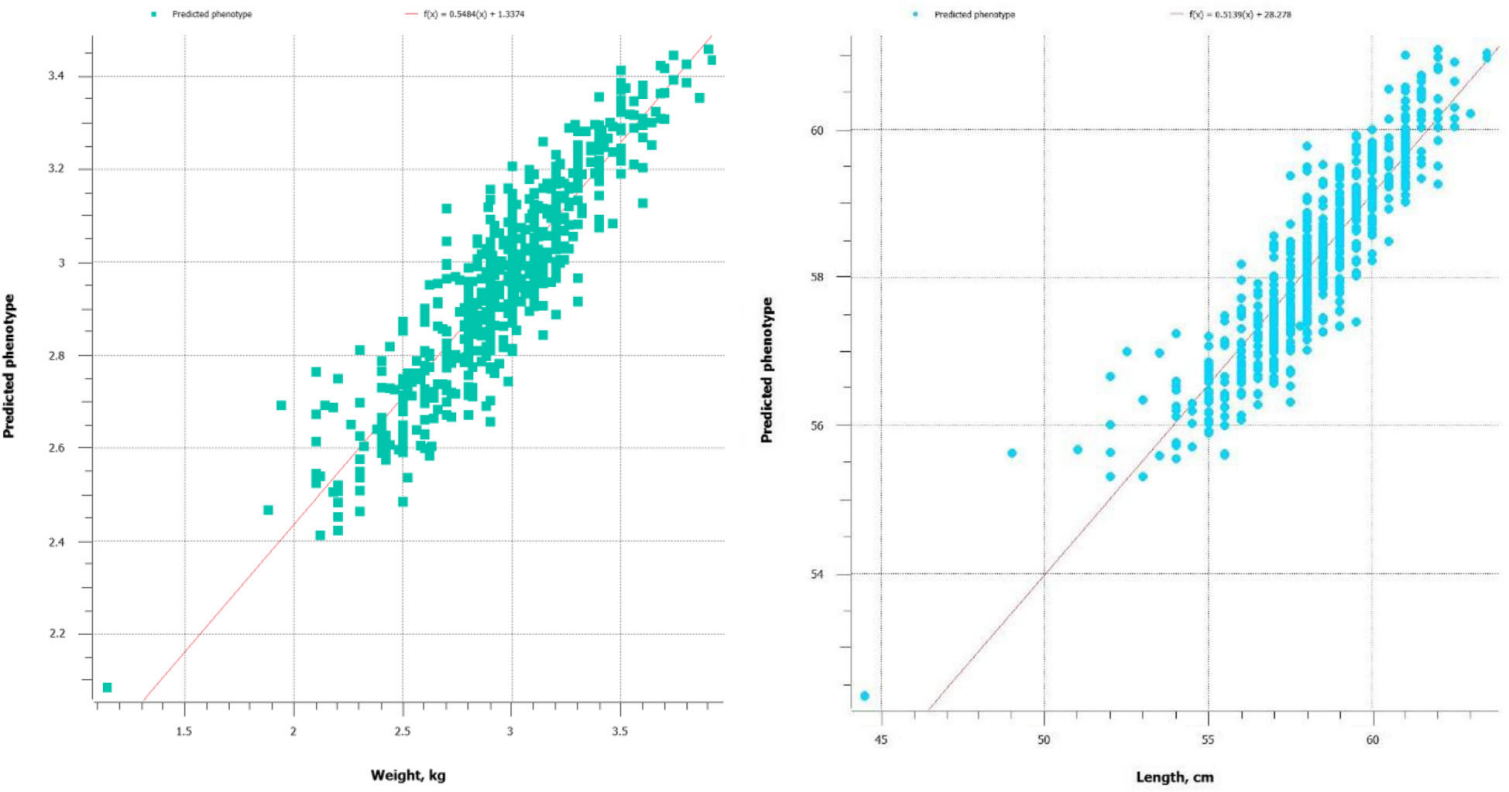
The predictive ability of gBLUP model for body weight and folk length (the correlation between actual and predicted phenotype *r* = 0.83 and 0.72, respectively), using imputed genotype data

The proportion of missing genotype was only about 14% in this population; however, imputations were made to have a complete genotype (without missing value). The imputation slightly increased predictive ability of genomic prediction for growth related traits (Fig. 2 vs. Table 2). For body weight, the predictive ability increased from 0.69 to 0.83 when analysis of the imputed data was compared with the original genotype provided by DArT.

### Effect of markers sub-sets

**Fig. 3:**
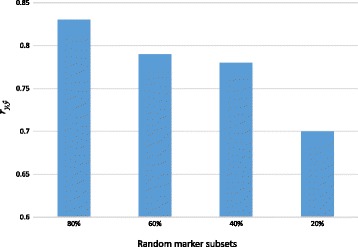
The predictive ability of gBLUP model for body weight using different random subsets of markers (20, 40, 60 and 90%)

Figure 3 shows the predictive ability of gBLUP model for body weight when different marker sub-sets randomly sampled from the dataset (20%, 40, 60% and 80% of 14,448 SNPs) were analysed. The predictive ability of the gBLUP model decreased when random marker subsets were used. The reduction in the predictive ability ranged from 13 to 18% across important traits studied. A graphical presentation of the predictive ability using different marker subsets is given for body weight in Fig. 3.

## Discussion

The central theme of this study was to determine the predictive ability of genomic prediction for traits of economic importance to enable the application of genomic selection in yellowtail kingfish (YTK). Our gBLUP analysis shows that the predictive ability obtained for growth-related traits (body weight, fork length and condition index) was moderate to high (0.44 to 0.69). A similar predictive ability was observed for body traits when numerator relationship matrix obtained from the pedigree was included in analyses. A linear regression analysis of the actual on predicted phenotypes also indicated a high predictive capacity of our models for the observed phenotype. The findings are particularly significant because one of the promising features of genomic selection is to reduce efforts of phenotyping in selection populations, and the performance of unphenotyped animals can be predicted from the training and validation populations from which they are derived. Our prediction within the same population using five-fold across validation approach also confirmed that there was a high correlation between the training and validation datasets for body weight, ${r}_{y,\widehat{y}}$ = 0.89.

Overall our results are in line with other studies reported in the literature that based on computer simulations or using empirical data from practical genetic enhancement breeding programs in farmed animals [26] or aquaculture species [15, 27–29]. However, comparing the results from different studies and populations is not rigorous due to differences in genetic background and histories of the experimental animals. The predictive ability of statistical methods used for genomic selection generally depends on marker effects, frequency of rare allele in populations [30]. In the present study, we found that gBLUP had similar predictive ability to Bayesian methods (i.e. Bayes C or Bayes Cπ, results were omitted). This is as expected for growth related traits that are well known under control by many genes, each with small effects. With regard to commercial application, gBLUP is preferred to other methods because it was less computationally demanding. Furthermore, gBLUP can account for various variance structure (e.g. heterogeneous variance due to genotype by environment interaction effect) and capture genetic relationship among individuals in the pedigree that are more important than capturing LD.

In the present study, we also found that the predictive ability of the gBLUP model is affected by the number of markers used in our analysis. A random sample of 20, 40, 60 and 80% of the total number of markers (14,448 SNPs) was analysed and the predictive ability of gBLUP model decreased by 13 to 18% across traits, in comparison with those obtained when the complete set of markers was used. In addition, we performed gBLUP analysis on a sub-set of 7988 markers after quality control. The marker filtering was based on two main criteria: i) < 10% missing values and 2) a minor allele frequency > 0.05. Linkage disequilibrium (LD) pruning was also applied to remove highly correlated markers. However, the predictive ability of the five-fold cross validation model for body traits was somewhat higher than those obtained from the full marker set (14,448 markers), e.g. ${r}_{y,\widehat{y}}$ = 0.90 for body weight.

Our five-fold across validation within the same population indicated very high predictive ability of the gBLUP model used especially for body weight. In this study, the full data that comprised 752 fish originating from the generation in 2014 were split into training and validation subsets. It is however necessary, in a future study, to sequence offspring of this population produced in the latest generation and re-train the model to ensure that capacity of gBLUP prediction is maintained before genomic selection can be considered as an option for future genetic improvement in this YTK population. The capacity of genomic prediction is likely to be reduced in subsequent generations, mainly as a result of recombination that causes a breakdown of the linkage disequilibrium (LD) between markers and quantitative trait loci (QTL). The prediction ability also depends on the number of distant ancestral generations included in the training set; the further generations apart the lower accuracy may be observed [31].

Collectively, the results achieved from this study showed the potential for genomic selection in this population of YTK. However, to enable the application of genomic selection in different populations (e.g. those from other States in Australia), genotyping a larger sample of animals would be needed for further prediction and validation. Genomic prediction equations developed for one population may not be applicable to another due to LD decays across generations as reported in farmed animals [32]. A larger training population together with a higher density SNP panel would also help to increase the accuracy of genomic prediction for traits with low heritability, namely skin fluke and deformity (results not tabulated). In dairy cattle, across-breed genomic selection used 300,000 SNPs and the accuracies of prediction ranged from 0.6 to 0.8 for economically important traits such as milk yield and fertility [33]. Whole genome sequencing also increased predictive power of genomic prediction, although the improvement was not large and varied with traits and studies [34]. Further, continuation of collecting more genomic data in future generations of this YTK population should be conducted to improve the reliability of genomic prediction for traits used here.

This study is the first reporting genomic prediction in marine yellowtail kingfish. Earlier studies involved computer simulation and showed potential benefits of genomic selection for aquatic animal species [35]. Nielsen et al. [36] demonstrated that the accuracy of genomic selection in a sib based breeding program can be 33% higher than selection based on conventional BLUP approach and genetic gain under optimal contribution selection based on genomic EBV can be improved by 81%. Implementation of genomic selection has been shown to reduce generation time, lower inbreeding and improve genetic gain for traits of commercial importance in agricultural species, e.g. in dairy cattle [37]. Recent studies in salmonids reported that the accuracy of genomic prediction ranged from 0.34 to 0.61 for growth traits [27] and from 0.45 to 0.71 for resistance to a range of diseases such as sea louse *Caligus rogercresseyi* [28], *Piscirickettsia* [29] or *Flavobacterium psychrophilum* [15]. A synthesised result from the literature together with our estimates obtained in this study suggest that genomic selection has the potential to accelerate genetic gain in the future breeding program for yellowtail kingfish. To improve the cost efficiency of genomic selection for this population, reduced sequence coverage in combination with imputation can be leveraged to genotype a larger number of training individuals to improve prediction accuracy and a larger number of selection candidates (validation population) to increase selection intensity and as a consequence, this can lead to increased genetic gain in YTK. A recent simulation study in plants [38] demonstrated that imputation can allow a reduced sequence coverage to as low as 1*×* with 10,000 markers and this can provide comparable genomic prediction accuracy as SNP arrays. Improved accuracy with imputation from a low- (256 SNPs) to high-density panel (250 K SNPs) is also shown in Atlantic salmon [39]. Return on investment from the low-cost genotyping by sequencing and imputation to enable a cost-effective genomic selection program merits future study in this species.

## Conclusions

Main findings from our first genotyping by sequencing study in yellowtail kingfish include: i) a large number of markers were derived from DArTseq™ sequencing technology and they can be used for genomic prediction and selection, ii) the predictive ability of gBLUP model for growth-related traits (weight, length and condition index) was high to enable the application of genome-based selection in this population, and iii) the markers subsets (e.g. 80% of the full set used in this study) can provide a comparable predictive capacity for growth characters. However, genomic prediction for traits with low heritability (e.g. skin fluke or deformity, results omitted) using genotyping by sequencing technology and imputation deserves further study in this population of yellowtail kingfish. A large scale routine data recording from multiple generations in in-depth pedigree populations is also needed to improve accuracy of genomic breeding value estimation for other traits of commercial importance in this species *S. lalandi*.

## Additional files

Additional file 1: Basic statistics of the sequence data used. Descriptive population genetic estimates and statistics of Single Nucleotide Polymorphisms (SNPs). (DOCX 27 kb)

## Abbreviations

DArT: Diversity Arrays Technology
EBV: Estimated Breeding Values
gBLUP: Genomic best linear unbiased prediction
GBS: Genotyping by Sequencing
SNP: Single Nucleotide Polymorphism

## References

[1] Symonds J, Walker S, Pether S, Gublin Y, McQueen D, King A, Irvine G, Setiawan A, Forsythe J, Bruce M (2014). Developing yellowtail kingfish (Seriola lalandi) and hāpuku (Polyprion oxygeneios) for New Zealand aquaculture. N Z J Mar Freshw Res.

[2] Knibb W, Miller A, Quinn J, D'Antignana T, Nguyen NH (2016). Comparison of lines shows selection response in kingfish (Seriola lalandi). Aquaculture.

[3] Whatmore P, Nguyen NH, Miller A, Lamont R, Powell D, D'Antignana T, Bubner E, Elizur A, Knibb W (2013). Genetic parameters for economically important traits in yellowtail kingfish *Seriola lalandi*. Aquaculture.

[4] Hung D, Vu NT, Nguyen NH, Ponzoni RW, Hurwood DA, Mather PB (2013). Genetic response to combined family selection for improved mean harvest weight in giant freshwater prawn (*Macrobrachium rosenbergii*) in Vietnam. Aquaculture.

[5] Dong Z, Nguyen NH, Zhu W (2015). Genetic evaluation of a selective breeding program for common carp *Cyprinus carpio* conducted from 2004 to 2014. BMC Genet.

[6] Oliveira CAL, Ribeiro RP, Yoshida GM, Kunita NM, Rizzato GS, Oliveira SN, Santos AI, Nguyen NH (2016). Correlated changes in body shape after five generations of selection to improve growth rate in a breeding program for Nile tilapia Oreochromis niloticus in Brazil. J Appl Genet.

[7] Hamzah A, Ponzoni RW, Nguyen NH, Khaw HL, Yee HY, Nor SAM (2014). Genetic evaluation of the Genetically Improved Farmed Tilapia (GIFT) strain over ten generations of selection in Malaysia. J Trop Agric Sci.

[8] Nguyen HN (2016). Genetic improvement for important farmed aquaculture species with a reference to carp, tilapia and prawns in Asia: achievements, lessons and challenges. Fish Fish.

[9] Thoa NP, Ninh NH, Knibb W, Nguyen NH (2016). Does selection in a challenging environment produce Nile tilapia genotypes that can thrive in a range of production systems?. Sci Rep.

[10] Kilian A, Wenzl P, Huttner E, Carling J, Xia L, Blois H, Caig V, Heller-Uszynska K, Jaccoud D, Hopper C (2012). Diversity arrays technology: a generic genome profiling technology on open platforms. Methods Mol Biol.

[11] Courtois B, Audebert A, Dardou A, Roques S, Ghneim-Herrera T, Droc G, Frouin J, Rouan L, Gozé E, Kilian A (2013). Genome-wide association mapping of root traits in a japonica rice panel. PLoS One.

[12] Von Mark VC, Kilian A, Dierig DA (2013). Development of DArT marker platforms and genetic diversity assessment of the US collection of the new oilseed crop lesquerella and related species. PLoS One.

[13] Raman H, Raman R, Kilian A, Detering F, Carling J, Coombes N, Diffey S, Kadkol G, Edwards D, McCully M (2014). Genome-wide delineation of natural variation for pod shatter resistance in *Brassica napus*. PLoS ONE.

[14] Tsai H-Y, Hamilton A, Tinch AE, Guy DR, Bron JE, Taggart JB, Gharbi K, Stear M, Matika O, Pong-Wong R (2016). Genomic prediction of host resistance to sea lice in farmed Atlantic salmon populations. Genet Sel Evol.

[15] Vallejo RL, Leeds TD, Gao G, Parsons JE, Martin KE, Evenhuis JP, Fragomeni BO, Wiens GD, Palti Y (2017). Genomic selection models double the accuracy of predicted breeding values for bacterial cold water disease resistance compared to a traditional pedigree-based model in rainbow trout aquaculture. Genet Sel Evol.

[16] Palaiokostas C, Ferraresso S, Franch R, Houston RD, Bargelloni L (2016). Genomic prediction of resistance to pasteurellosis in gilthead sea bream (Sparus aurata) using 2b-RAD sequencing. G3.

[17] Nguyen N, Whatmore P, Miller A, Knibb W (2016). Quantitative genetic properties of four measures of deformity in yellowtail kingfish Seriola lalandi Valenciennes, 1833. J Fish Dis.

[18] Renshaw MA, Patton JC, Rexroad CE, Gold JR (2006). Isolation and characterization of dinucleotide microsatellites in greater amberjack, Seriola dumerili. Conserv Genet.

[19] Renshaw MA, Patton JC, Rexroad CE, Gold JR (2006). PCR primers for trinucleotide and tetranucleotide microsatellites in greater amberjack, Seriola dumerili. Mol Ecol Notes.

[20] Jones OR, Wang J (2010). Molecular marker-based pedigrees for animal conservation biologists. Anim Conserv.

[21] Elshire RJ, Glaubitz JC, Sun Q, Poland JA, Kawamoto K, Buckler ES, Mitchell SE (2011). A robust, simple genotyping-by-sequencing (GBS) approach for high diversity species. PloS one..

[22] Gilmour AR, Gogel B, Cullis B, Thompson R, Butler D (2009). ASReml user guide release 3.0.

[23] de los Campos G, Hickey JM, Pong-Wong R, Daetwyler HD, Calus MP (2013). Whole-genome regression and prediction methods applied to plant and animal breeding. Genetics.

[24] Clark SA, Hickey JM, Van der Werf JH (2011). Different models of genetic variation and their effect on genomic evaluation. Genet Sel Evol.

[25] VanRaden P, Van Tassell C, Wiggans G, Sonstegard T, Schnabel R, Taylor J, Schenkel F (2009). Invited review: reliability of genomic predictions for North American Holstein bulls. J Dairy Sci.

[26] Daetwyler HD, Calus MP, Pong-Wong R, de los Campos G, Hickey JM. Genomic prediction in animals and plants: simulation of data, validation, reporting, and benchmarking. Genetics. 2013;193(2):347-65. 10.1534/genetics.112.147983 PMC3567728 23222650

[27] Tsai HY, Hamilton A, Tinch AE, Guy DR, Gharbi K, Stear MJ (2015). Genome wide association and genomic prediction for growth traits in juvenile farmed Atlantic salmon using a high density SNP array. BMC Genomics.

[28] Correa K, Bangera R, Figueroa R, Lhorente JP, Yáñez JM (2017). The use of genomic information increases the accuracy of breeding value predictions for sea louse (Caligus rogercresseyi) resistance in Atlantic salmon (Salmo salar). Genet Sel Evol.

[29] Bangera R, Correa K, Lhorente JP, Figueroa R, Yáñez JM (2017). Genomic predictions can accelerate selection for resistance against Piscirickettsia salmonis in Atlantic salmon (Salmo salar). BMC Genomics.

[30] Goddard M (2009). Genomic selection: prediction of accuracy and maximisation of long term response. Genetica.

[31] Weng Z, Wolc A, Shen X, Fernando RL, Dekkers JCM, Arango J, Settar P, Fulton JE, O’Sullivan NP, Garrick DJ (2016). Effects of number of training generations on genomic prediction for various traits in a layer chicken population. Genet Sel Evol.

[32] Hayes B, Bowman P, Chamberlain A, Verbyla K, Goddard M (2009). Accuracy of genomic breeding values in multi-breed dairy cattle populations. Genet Sel Evol.

[33] van Binsbergen R, Calus MP, Bink MC, van Eeuwijk FA, Schrooten C, Veerkamp RF (2015). Genomic prediction using imputed whole-genome sequence data in Holstein Friesian cattle. Genet Sel Evol.

[34] Iheshiulor OO, Woolliams JA, Yu X, Wellmann R, Meuwissen TH (2016). Within-and across-breed genomic prediction using whole-genome sequence and single nucleotide polymorphism panels. Genet Sel Evol.

[35] Sonesson A, Meuwissen T (2009). Testing strategies for genomic selection in aquaculture breeding programs. Genet Sel Evol.

[36] Nielsen H, Sonesson A, Yazdi H, Meuwissen T (2009). Comparison of accuracy of genome-wide and BLUP breeding value estimates in sib based aquaculture breeding schemes. Aquaculture.

[37] García-Ruiz A, Cole JB, VanRaden PM, Wiggans GR, Ruiz-López FJ, Van Tassell CP (2016). Changes in genetic selection differentials and generation intervals in US Holstein dairy cattle as a result of genomic selection. Proc Nat Acad Sci.

[38] Gorjanc G, Dumasy J-F, Gonen S, Gaynor RC, Antolin R, Hickey JM (2017). Potential of low-coverage genotyping-by-sequencing and imputation for cost-effective genomic selection in biparental segregating populations. Crop Sci.

[39] Tsai H-Y, Matika O, Edwards SM, Antolín–Sánchez R, Hamilton A, Guy DR, Tinch AE, Gharbi K, Stear MJ, Taggart JB (2017). Genotype imputation to improve the cost-efficiency of genomic selection in farmed Atlantic salmon. G3.

