# Supplementary material for: Genomic prediction using DArT-Seq technology for yellowtail kingfish Seriola lalandi
Source: BMC Genomics. 2018 Jan 30;19:107. doi: 10.1186/s12864-018-4493-4 (PMC5791361; doi:10.1186/s12864-018-4493-4)
Supplement: Supplementary file 1 — Basic statistics of the sequence data used. Descriptive population genetic estimates and statistics of Single Nucleotide Polymorphisms (SNPs). (DOCX 27 kb) [file 12864_2018_4493_MOESM1_ESM.docx]

**Genomic prediction using DArT-Seq technology for yellowtail kingfish *Seriola lalandi***

Nguyen H Nguyen^1,^*, Ajith Premachandra^1^, Andrzej Kilian^2^ and Wayne Knibb^1^

^1^The University of the Sunshine Coast, Maroochydore DC, QLD 4558, Australia

^2^Diversity Arrays Technology Pty Ltd, Kirinari St. Bruce, ACT2617, Australia

***Correspondence to** [**NNguyen@usc.edu.au**](mailto:NNguyen@usc.edu.au)

Table S1. Basic statistics of the sequence data used.

| **SNP statistics** | Mean | Range |
| --- | --- | --- |
| Total SNPs | 14,448 |  |
| AvgReadDepth | 614.8 | 3 – 363 |
| AvgCountSnp | 25.9 | 2 – 252 |
| Call rate | 0.92 | 0.39 – 1 |
| OneRatioSnp (% samples for which SNP = 1) | 0.25 | 0 – 1 |
| FreqHomSnp (%samples which score homozygote) | 0.16 | 0 – 1 |
| FreqHets (%samples which score heterozygote) | 0.09 | 0 – 1 |
| PIC SNP | 0.16 | 0 – 0.5 |

| Call rate | The proportion of samples for which the genotype call is either '1' or '0', rather than '-'. |
| --- | --- |
| OneRatioSNP | The proportion of samples for which the genotype score is '1', in the SNP allele row. |
| FredHomSNP | The proprtion of samples which score as homozygous for the SNP allele. |
| FredHets | The proprtion of samples which score as heterozygous. |
| PIC SNP | The polymorphism information content (PIC) of the SNP allele row. |
| AvgCountSNP | The sum of the tag read counts for all samples, divided by the number of samples with non-zero tag read counts, for the SNP allele row. |

Table S2: Descriptive population genetic estimates and statistics of single nucleotide polymorphisms (SNPs)

| Markers statistics | Mean | Minimum | Maximum |
| --- | --- | --- | --- |
| Call Rate | 0.98 | 0.9 | 1 |
| Minor Allele Frequency | 0.29 | 0.05 | 0.5 |
| Major Allele Frequency | 0.71 | 0.5 | 0.95 |
| Expected HWE P | 0.5 | 0 | 1 |
| Minor Allele Count | 428.57 | 72 | 752 |
| Major Allele Count | 1045.87 | 678 | 1428 |
| Missing Allele Count | 29.56 | 0 | 150 |
